# Mortality in Cyprus Over the Period 2016-2021

**DOI:** 10.7759/cureus.24325

**Published:** 2022-04-20

**Authors:** Demetris Avraam, Eleftheria C Economidou, Jannis Kountouras, Michael Doulberis, Elpidoforos S Soteriades

**Affiliations:** 1 Population Health Sciences Institute, Newcastle University, Newcastle, GBR; 2 Department of Public Health, University of Copenhagen, Copenhagen, DNK; 3 School of Medicine, University of Ioannina, Ioannina, GRC; 4 Department of Internal Medicine, Second Medical Clinic, Ippokration Hospital, Aristotle University of Thessaloniki, Thessaloniki, GRC; 5 Division of Gastroenterology and Hepatology, Medical University Department, Kantonsspital Aarau, Aarau, CHE; 6 Healthcare Management Program, School of Economics and Management, Open University of Cyprus, Nicosia, CYP; 7 Department of Environmental Health, Environmental and Occupational Medicine and Epidemiology, Harvard T.H. Chan School of Public Health, Boston, USA

**Keywords:** epidemic, covid-19, cyprus, mortality, excess deaths

## Abstract

Background

Mortality in the general population is one of the most robust measures used to examine epidemiological trends over time and especially over periods of public health crises such as the current coronavirus disease 2019 (COVID-19) pandemic.

Methodology

In this study, we analyzed information reported by the Cyprus Ministry of Health to the European Statistical Office (Eurostat), which includes weekly all-cause mortality over the period 2016-2021. In addition, we used data collected by the European Centre for Disease Prevention and Control regarding daily reported COVID-19 cases and COVID-19-related deaths.

Results

Based on our data analysis, we observed a substantial increase of 9.7% in all-cause mortality in Cyprus in 2021 compared to 2020, with an overall mortality increase of 16.5% in 2021 compared to the mean mortality of the previous five years. Particularly, we documented a sharp increase over the third and the fourth quarters of the year 2021.

Conclusions

The substantial increase in mortality in Cyprus in 2021 is not entirely explained by COVID-19 deaths and is parallel to the concurrent vaccination campaign. This concerning observation should be comprehensively investigated by the National and European public health authorities to identify and address the underlying causes.

## Introduction

Policymakers require concrete and reliable data regarding disease burden to efficiently allocate resources at the population level including vulnerable groups and evaluate public health interventions [1]. Public health depends on information derived from monitoring population health status to identify, diagnose, and investigate health problems and health hazards in the community. Through different stages of the evolution of the healthcare system, there have been close relations between health policy and strategy, the overall national development efforts, and the wider social and economic environment. Awareness of these relations is valuable in the understanding of current health issues such as the lifestyle-related patterns of morbidity and mortality, characterized by non-communicable and infectious diseases, the high demands for healthcare services, and evolving associations between the public and private sectors. Specifically, the scientific community appears to play a critical role in advancing the methods to support vigorous measurements of health-­related sustainable development goals [2]. In this respect, relative vital statistics constitute important systematic reporting systems [3].

The coronavirus disease 2019 (COVID-19) pandemic has triggered tremendous interest in related statistics. Therefore, in April 2020, in cooperation with the national statistical institutes of the European Statistical System, Eurostat, set up a special data collection on weekly deaths to support the policy and research efforts related to the pandemic. National statistical institutes regularly and voluntarily report data to Eurostat on weekly fatalities [4]. Population statistics are essential for epidemiological surveillance including morbidity and mortality. Such measures describe the progression and severity of health events. They are useful tools to study disease risk factors as well as to compare and contrast health events within and between different populations and time periods. Morbidity is the state of being symptomatic or unhealthy in relation to a disease or condition, whereas mortality or death rate is the number of deaths in a specific population in relation to the whole population (size of the population) for a specified period of time. It can be communicated as a rate or as an absolute number [5]. Such statistics have received heightened attention during the past couple of years owing to the COVID-19 pandemic. The National Center for Health Statistics (NCHS) monitors and reports deaths in the context of COVID-19 [6]. The ongoing COVID-19 pandemic constitutes a new challenge for public health and has resulted in numerous negative health-related consequences, including impacts on deaths. Particularly, the emergence and rapid worldwide spread of the Delta and, more recently, Omicron variants of severe acute respiratory syndrome coronavirus 2 (SARS-CoV-2) constitute a discouraging public health emergency. Public health professionals are faced with many pressures as COVID-19 increases the urgency and scope of their work and reduces their capacity to carry out core services [7]. During public health emergencies, the provision of regular public health services can be interrupted [8]; and depending on the type and scope of the emergency and the size and capability of the public health workforce, the response may represent a burden that negatively affects the effectiveness of reducing COVID-19-related mortality. Mortality data from the National Vital Statistics System are a primary source of information for identifying and monitoring acute and chronic diseases and other public health problems [9]. Mortality in the general population is one of the most robust measures used to track epidemiological trends over time and is being followed by governments and scientists alike with particular attention during the current epidemic [10].

Cyprus, along with the rest of the world, is currently undergoing the fourth wave of the COVID-19 epidemic [11]. From January 03, 2020, until December 31, 2021, there have been 161,779 confirmed cases of COVID-19 with 641 deaths in Cyprus reported to the World Health Organization (WHO) [12]. Several global reports are being focused on morbidity and mortality associated with SARS-CoV-2 [5]. However, one of the most important epidemiological indicators to assess population health is all-cause mortality [13]. Aiming to examine the potential impact of the epidemic on vital statistics, particularly mortality in the general population, we examined all-cause mortality in Cyprus during the six-year period from 2016 to 2021.

## Materials and methods

To analyze mortality data from Cyprus, we used the official data reported by the Cyprus Ministry of Health to the mentioned European Statistical Office (Eurostat), which included the weekly number of all-cause deaths [14]. From the analysis, we excluded the 53rd week of the year 2020 (December 28, 2020, to January 03, 2021) in the absence of a corresponding week in the other five years under examination. Note that the weeks are specified by the ISO (International Organization for Standardization) week-numbering system which means that each week starts on Monday and ends on Sunday (for example the first week of 2021 started on January 04 and ended on January 10). We also used data collected by the European Centre for Disease Prevention and Control on a daily basis regarding COVID-19-positive cases and COVID-19-related deaths [15].

Statistical analyses were performed using the open-source statistical software R version 4.0.4 [16]. Descriptive statistics and t-tests were used to compare mortality over time and between years. We have also applied two-sample t-tests to check whether the changes in the average weekly number of deaths for each of the two consecutive years are statistically significant for a significance level of 0.05.

We have tested the following hypothesis: null hypothesis: $m_t=m_{t+1}$ (i.e., true difference in means is equal to 0); alternative hypothesis: $m_t\neq m_{t+1}$ (i.e., true difference in means is not equal to 0), where $m_t$ is the average of weekly number of deaths during year $t$ (or during a specific quarter of year $t$).

## Results

**Figure 1:**
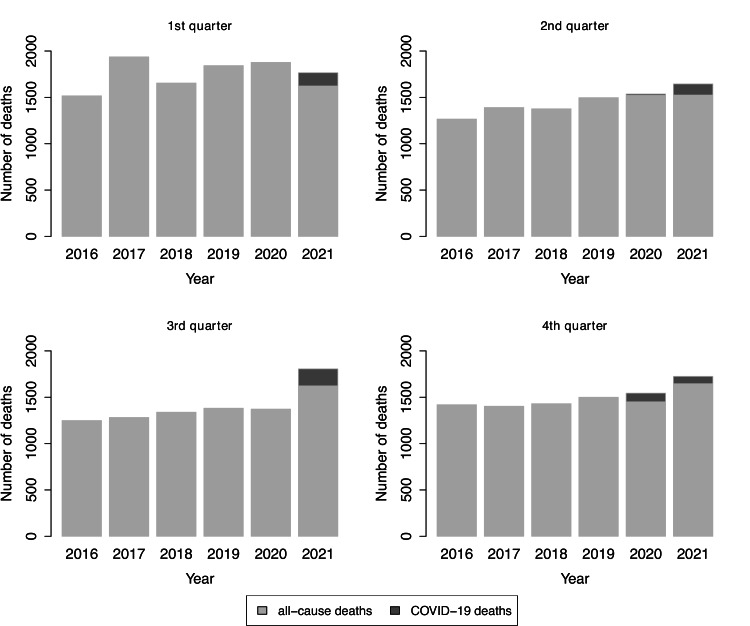
Number of all-cause deaths over time in Cyprus for the period 2016-2021. The figure illustrates the number of all-cause deaths during the first quarter (top-left panel), second quarter (top-right panel), third quarter (bottom-left panel), and fourth quarter (bottom-right panel) of each year. The upper level of each bar indicates the total number of deaths from all causes while the darker gray in the years 2020 and 2021 indicates the proportion of COVID-19 deaths. COVID-19: coronavirus disease 2019

During the year 2021, a total of 6,942 all-cause deaths were reported in Cyprus compared to 6,329 all-cause deaths reported in 2020, the first year of the COVID-19 pandemic [14]. This difference represents a cumulative increase of 9.7%. A reasonable concern arises from this worrying increase, that is, whether it is all explained by the number of deaths that were reported due to COVID-19 during the same time period. COVID-19 deaths over the year 2021 in Cyprus were 525, while for the year 2020 were 115 (note that the first COVID-19 death in Cyprus was reported on March 21, 2020, the 12th week of the year) [15]. Furthermore, by excluding COVID-19 deaths from the total number of deaths, we observed an increase of 3.3% in mortality from 2020 to 2021. The increase of 9.7% in all-cause mortality from 2020 to 2021 was not all explained by COVID-19 deaths. In addition, by calculating the percentage change of deaths for each of the two consecutive years over the last six years (from 2016 to 2021), we observed that the increase in mortality was not a part of an expected trend over time. Figure 1 displays the number of all-cause deaths in Cyprus from 2016 up to 2021.

Specifically, detailed comparisons for each quarter of the years under study are shown in Figure 1. The top-left panel of Figure 1 indicates that over the first quarter of 2021, the total number of all-cause deaths was lower than the total number of all-cause deaths over the same quarter of 2020 (1,766 deaths in 2021 compared to 1,877 in 2020) [14], with 143 cases reported as COVID-19 deaths in the first quarter of 2021 compared to four COVID-19 deaths during the same quarter in 2020. Likewise, the number of deaths in the first quarter of 2021 was within one standard deviation from the mean of deaths in the first quarters of the years 2016-2020 ($\mu=1,765, \sigma=174.7$).

The top-right panel of Figure 1 displays a significant increase in the number of all-cause deaths over the second quarter of 2021 compared to the same quarter of 2020. A total of 1,645 deaths were reported during the second quarter of 2021 compared to 1,536 deaths reported in the same quarter of 2020 [14]. However, this difference is well explained by the 105 more COVID-19 deaths reported in the second quarter of 2021 compared to the same quarter of 2020 (15 and 120 COVID-19 deaths in the second quarter of 2020 and 2021, respectively). The number of all-cause deaths in the second quarter of 2021 was within the interval of three standard deviations from the mean of deaths of the second quarters of the years 2016-2020 ($\mu=1,412.6, \sigma=107$).

The bottom-left panel of Figure 1 displays a considerable increase in all-cause mortality during the third quarter of 2021 compared with the number of all-cause deaths during the same quarter of the previous five years. During the third quarter of 2021, 1,806 deaths were reported, 434 excess deaths compared to 1,372 deaths during the same quarter in 2020 [14]. When excluding deaths attributed to COVID-19, the number of all-cause deaths in the third quarter of 2021 was 1,623, which equaled to 254 excess deaths compared to the 1,369 comparable deaths in the third quarter of 2020. Moreover, the number of all-cause deaths in the third quarter of 2021 was more than eight standard deviations further from the mean of deaths in the third quarters of the years 2016-2020 ($\mu=1,324, \sigma=57.8$).

The bottom-right panel of Figure 1 displays the increase in all-cause deaths during the fourth quarter of 2021 compared to the same quarter of the previous five years. During the fourth quarter of 2021, 1,725 deaths were reported resulting in 181 more deaths compared to the 1,544 deaths reported during the fourth quarter of 2020 or 266 more deaths compared to the mean of 1,459 deaths during the first quarters of the previous five years (2016-2020) [14]. When excluding the number of COVID-19 deaths from this comparison [15], we observed 195 more (non-COVID-19) deaths in 2021 compared to 2020.

The analytical results of the above comparisons are displayed in Table 1, which also shows the total number of deaths from all causes and the mean weekly deaths as well as the percentage change for each of the two consecutive years. Table 1 indicates a substantial increase in mortality in the general population of Cyprus during the second, third, and fourth quarters of 2021 compared to last year (5.9% decrease in the first quarter, 7.1% increase in the second quarter, 31.6% increase in the third quarter, and 11.7% increase in the fourth quarter). The table also shows the difference in means of the weekly number of deaths over the third and the fourth quarters between the years 2020 and 2021 was statistically significant (p < 0.05).

**Table 1:** Mortality in Cyprus by quarter including total and mean weekly deaths and percentage change for each of the two consecutive years over the time period 2016-2021.

Year	First quarter				Second quarter				Third quarter				Fourth quarter			
	Total deaths	% change	Mean weekly deaths	P-value	Total deaths	% change	Mean weekly deaths	P-value	Total deaths	% change	Mean weekly deaths	P-value	Total deaths	% change	Mean weekly deaths	P-value
2016	1,516		116.6		1,265		97.3		1,248		96.0		1,419		109.2	
2017	1,936	+27.7	148.9	<0.05	1,390	+9.9	106.9	0.08	1,281	+2.6	98.5	0.61	1,402	-1.2	107.8	0.82
2018	1,654	-14.6	127.2	<0.05	1,376	-1.0	105.8	0.84	1,338	+4.4	102.9	0.39	1,430	+2.0	110.0	0.61
2019	1,842	+11.4	141.7	<0.05	1,496	+8.7	115.1	0.12	1,381	+3.2	106.2	0.53	1,499	+4.8	115.3	0.32
2020	1,877	+1.9	144.4	0.68	1,536	+2.7	118.2	0.69	1,372	-0.7	105.5	0.90	1,544	+3.0	118.8	0.55
2021	1,766	-5.9	135.8	0.21	1,645	+7.1	126.5	0.35	1,806	+31.6	138.9	<0.05	1,725	+11.7	132.7	<0.05

## Discussion

In this study, we analyzed all-cause mortality data from Cyprus over the last six years (2016-2021) and observed a substantial increase of 9.7% in 2021 compared to 2020 [17]. Thus, a reasonable concern arises from this worrying increase, whether it is all explained by the number of deaths that were reported due to COVID-19 during the same period. Moreover, we found that, when excluding the deaths reported to be caused by the COVID-19 disease [18], there is a 3.3% increase in the mortality of the general population. Therefore, the worrying increase of 9.7% of all-cause mortality in 2021 does not appear to be explained entirely by COVID-19 deaths.

Overall, the findings of our analysis are worrying regarding the year 2021, demonstrating a substantial, statistically significant, elevation in mortality from all causes in the general population, especially during the third and the fourth quarters of 2021. At this stage, in the absence of granular data, we are not able to attribute the increase to specific causes. However, excluding the reported number of deaths from COVID-19, we observed an increase in the total mortality from all causes in Cyprus, especially in the third and the fourth quarters of 2021, based on data released by Eurostat, a phenomenon which coincides chronologically with the parallel vaccination campaign. Owing to these worrying concerns, future relative research is warranted to elucidate in-depth knowledge regarding these critical issues not only on a national but also on a global level.

Meanwhile, we expect that the public health authorities in Cyprus, including the Ministry of Health and the Cyprus Medical Association, will dig into these data to perform a detailed and analytical investigation of this sharp increase in an effort to identify the underlying causes. Detailed information about all individual-level cases, such as the age, gender, geographical distribution, medical history, vaccination status, cause of death, and seasonality, are necessary to scientifically elucidate the hidden etiologies [19].

## Conclusions

Excess mortality in Europe has been identified as one of the most useful indicators for assessing additional deaths, complementing the other indicators followed by the European Statistical Recovery Dashboard. To capture the dynamics of mortality in a more stable way, the excess mortality indicator is calculated for each month (depending on data available to Eurostat from the national statistical institutes). The indicator provides additional insight into the impact that the COVID-19 crisis has exerted on European societies. Based on our findings, we believe that it is crucial that similar studies at the European level are also conducted and timely evaluated.
